# To Subscribers, Correspondents, &c.

**Published:** 1859-11

**Authors:** 


**To Subscribers, Correspondents, &c.**

By a recent change in the management of the Journal, its whole business affairs will hereafter come under the supervision of the Senior Editor, and it is therefore requested that *all* communications, whether relating to the Editorial, Subscription, Advertising, or Financial Departments, should be addressed to Jos. P. Logan, or Editors of Medical and Surgical Journal, Atlanta, Ga.

